# Intermediate predator naïveté and sex-skewed vulnerability predict the impact of an invasive higher predator

**DOI:** 10.1038/s41598-018-32728-0

**Published:** 2018-09-24

**Authors:** Ross N. Cuthbert, Tatenda Dalu, Ryan J. Wasserman, Jaimie T. A. Dick, Lubabalo Mofu, Amanda Callaghan, Olaf L. F. Weyl

**Affiliations:** 1 Institute for Global Food Security, School of Biological Sciences, Medical Biology Centre, Queen’s University Belfast, Belfast, BT9 7BL Northern Ireland UK; 2 DST/NRF Research Chair in Inland Fisheries and Freshwater Ecology, South African Institute for Aquatic Biodiversity (SAIAB), Grahamstown, 6140 South Africa; 3 Ecology and Evolutionary Biology, School of Biological Sciences, University of Reading, Harborne Building, Reading, RG6 6AS England UK; 4 Department of Ecology and Resource Management, University of Venda, Thohoyandou, 0950 South Africa; 5 Department of Biological Sciences and Biotechnology, Botswana International University of Science and Technology, P. Bag 16, Palapye, Botswana; 6 South African Institute for Aquatic Biodiversity (SAIAB), Grahamstown, 6140 South Africa

## Abstract

The spread of invasive species continues to reduce biodiversity across all regions and habitat types globally. However, invader impact prediction can be nebulous, and approaches often fail to integrate coupled direct and indirect invader effects. Here, we examine the ecological impacts of an invasive higher predator on lower trophic groups, further developing methodologies to more holistically quantify invader impact. We employ functional response (FR, resource use under different densities) and prey switching experiments to examine the trait- and density-mediated impacts of the invasive mosquitofish *Gambusia affinis* on an endemic intermediate predator *Lovenula raynerae* (Copepoda). *Lovenula raynerae* effectively consumed larval mosquitoes, but was naïve to mosquitofish cues, with attack rates and handling times of the intermediate predator unaffected by mosquitofish cue-treated water. Mosquitofish did not switch between male and female prey, consistently displaying a strong preference for female copepods. We thus demonstrate a lack of risk-reduction activity in the presence of invasive fish by *L. raynerae* and, in turn, high susceptibility of such intermediate trophic groups to invader impact. Further, we show that mosquitofish demonstrate sex-skewed predator selectivity towards intermediate predators of mosquito larvae, which may affect predator population demographics and, perversely, increase disease vector proliferations. We advocate the utility of FRs and prey switching combined to holistically quantify invasive species impact potential on native organisms at multiple trophic levels.

## Introduction

Invasive species incursions and proliferations are accelerating and present an enormous threat to environments and economies globally^1,2^. Freshwater ecosystems are particularly vulnerable to invasions due to high human-mediated propagule pressure and interconnectedness enabling rapid establishment and spread^3,4^. Indeed, anthropogenic modifications of freshwater systems, such as flow manipulation^5^ and impoundment construction^6^, can further heighten vulnerabilities to invaders^7,8^. Naïveté of native communities can exacerbate suppressive interactions with invasive species, especially in insular ecosystems (e.g. freshwaters) where there are no trophically analogous natives^9–12^. In particular, prey naïveté to unfamiliar cues or behaviours can profoundly increase impacts by invasive predators compared to native equivalents^13,14^. Reciprocally, naïveté can also influence biotic resistance between naïve native predators and invasive prey through processes such as prey preferences and switching with native prey^15–17^. However, invasion science has been slow to develop predictive methods to quantify invader impacts, and we currently lack quantitative approaches to forecast how prey naïveté and demography may affect invader impact strengths in recipient environments at multiple trophic levels.

Invasive fishes have been especially damaging to freshwater ecosystems, driving extinctions of indigenous species^18^. Human-mediated introductions of fish into novel, previously fishless systems risk fundamentally altering species compositions and diversities through processes such as predation^19,20^. A key challenge therefore surrounds the quantification and prediction of invasive higher predator impacts on underlying trophic groups. These impacts can be profound^21^, and may manifest in trophic cascades driven by both consumptive, density-mediated indirect interactions (DMIIs^22,23^), and non-consumptive, trait-mediated indirect interactions (TMIIs^23,24^). Critically, TMII effects may be as impactful as those resulting from direct consumption^24–26^. These effects can, in turn, be dependent on coevolutionary histories between trophic groups, or ‘adaptive lag’ of native assemblages^27^, and aquatic systems present an ideal platform to examine indirect, TMII effects due to the prevalence and ease of manipulation of water-borne predator cues^28^. However, predicting impacts by invasive species on native prey can be complicated due to density- and context- dependencies, which may be non-additive in effect^29–31^.

Functional responses (FRs) have been used extensively in the quantification of consumer-resource interactions, and FRs can be powerful tools to quantify density- and context-dependencies of invader impact^32–34^. Indeed, FRs can be applied to examine multiple predator effects between interacting con- and interspecific invasive species^21,30^. In the context of predation, the FR encapsulates prey consumption by predators in relation to prey density, with both FR form and magnitude powerful indicators for the derivation of consumer impact strengths^34^. Three common forms of FRs have been categorised: (1) Type I FRs are regarded as filter feeder-specific, with intake increasing linearly with prey availability^35^; (2) Type II FRs are characterised by a decelerating intake rate, which may be conducive to prey destabilisation as a result of high proportional consumption at low prey densities^34^; (3) Type III FRs are, in turn, characterised by low intake rate at low prey densities, and are sigmoidal in form^33^, thus potentially imparting stability to prey populations by facilitating refugia for prey at low densities. The application of comparative FRs can not only be informative in terms of relative consumer impacts, but also directly enables the derivation of emergent context-dependencies that modulate consumer-resource interaction strengths^34,36^. These effects can be both abiotic (e.g. temperature/structural complexity^37^) and biotic (e.g. higher predators^21^). For instance, the detection of kairomones from a familiar higher predator can modify foraging intensity of intermediate predators towards basal prey^38^. This may manifest in modulations to the form and magnitude of FRs^28^. However, in cases where an intermediate predator is exposed to a novel threat, these responses may be nullified due to naïveté and, thus, predation vulnerability may not be alleviated.

Another classic concept within consumer-resource ecology surrounds prey switching, or frequency-dependence of predation^39^. Prey switching may be a powerful indicator when utilised alongside FRs to examine consumptive traits and impacts. However, prey switching has hitherto remained under-applied in invasion science, reducing our capacity to predict invader impacts (but see Cuthbert *et al*.^17^). Characteristically, when consumers exhibit a prey switching propensity, disproportionately more of the abundant prey type are consumed whilst disproportionately fewer rare prey are consumed^39^. This can foster stability in diverse prey populations, enabling coexistence patterns to emerge. Indeed, prey switching can be a key driver of the sigmoidal, stabilising Type III FR^40^. Furthermore, switching between intraspecific prey types can have demographic implications, particularly if prey consumption is sex-skewed. In turn, this can lead to emergent inequalities in sex ratios which may affect the population persistence of lower trophic groups^41,42^. As such, quantifying prey switching propensities between intraspecific prey forms can elucidate likely demographic and density-mediated outcomes for prey species following novel higher predator introductions.

The mosquitofish, *Gambusia affinis* (Baird and Girard), is one of the most widespread fish globally, having been introduced extensively in mosquito control efforts in recent decades^43^. Further, it is regarded as one of the world’s worst invasive species^44^, inducing negative impacts on native fish, amphibians and aquatic invertebrates^20,45,46^. The effectiveness of mosquitofish in biological control has been fundamentally questioned^47^, and their application has been recorded to, perversely, increase mosquito proliferations due to interguild predation upon intermediate trophic groups such as notonectids^48^. In turn, this has resulted in calls to cease the use of such non-native fish in biological control efforts^49^. Furthermore, mosquitoes have been shown to comprise less than 1% of the diet of *G. affinis*, whilst zooplankton compose a majority^50^, demonstrating generalist feeding strategies that reduce biological control efficacy of the mosquitofish. Yet, we currently lack holistic impact quantifications of such invasive species upon ecosystems outside of their native range.

In the present study, we thus use FR and prey switching experiments to quantify the impact of *G. affinis* on native trophic groups which are vulnerable to localised extinctions^19^. We examine the responsiveness of an intermediate predator, endemic to South Africa, the open-water calanoid copepod *Lovenula raynerae* Suárez-Morales, Wasserman and Dalu to water-borne mosquitofish cues, using mosquito larvae of the *Culex pipiens* complex as a basal prey. The *C. pipiens* mosquito complex is widespread globally, and colonises an extensive range of aquatic habitats, including temporary ponds. Calanoid copepods are also widespread and form an abundant and important component of freshwater ecosystems^51^. *Lovenula raynerae* is an ephemeral pond specialist species^52^, and thus has evolved within fishless aquatic systems. Given a limited distribution, this copepod is highly vulnerable to environmental change. Indeed, mosquitofish have been documented to invade ephemeral systems^53,54^, and *L. raynerae* have been detected in longstanding fishless systems where fish may persist if introduced (Wasserman pers. obs.). Thus, the potential for impact of mosquitofish on such vulnerable populations is high. Our approach examines responsiveness of *L. raynerae* consumption to visual and chemical mosquitofish cues and thus naïveté to predation by the novel invader. Additionally, we examine prey switching propensities of mosquitofish between female and male copepods, elucidating whether predation of *L. raynerae* by *G. affinis* will affect prey population viability through the establishment of sex-skewed ratios. Thus, we aim to illustrate the likely trait- and density-mediated impacts of the introduction of an invader on an intermediate predator and the cascade to its prey.

## Results

Prey survival in controls exceeded 99% in both experiments, thus we assumed experimental deaths were due to predation, which we also observed directly. In Experiment 1, overall consumption by copepods was not significantly affected by the presence of *G. affinis* chemical cues (*χ*^2^ = 0.09, *df* = 1, *p* = 0.76), visual cues (*χ*^2^ = 0.02, *df* = 1, *p* = 0.88), or interaction between these cues (*χ*^2^ = 0.10, *df* = 1, *p* = 0.76). Overall prey consumption was significantly greater under increasing prey supplies (*χ*^2^ = 30.61, *df* = 4, *p* < 0.001). Further interactions among ‘chemical cue’, ‘visual cue’ and ‘prey supply’ were non-significant and thus were removed stepwise from the model.

**Figure 1:**
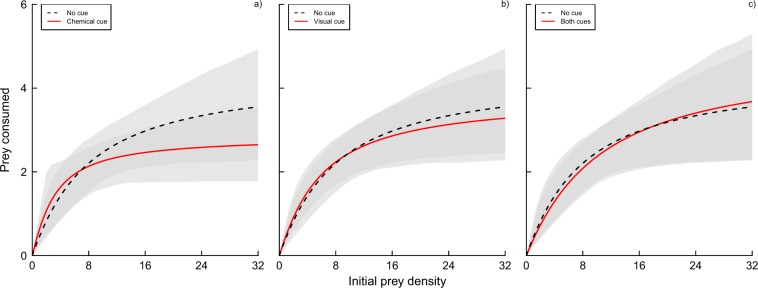
Functional responses of male *L. raynerae* towards larval culicid prey without cues of *G. affinis* compared to FRs in the presence of (**a**) chemical cues, (**b**) visual cues and (**c**) both cues. Shaded areas around FRs represent bootstrapped (*n* = 2000) confidence intervals.

As first order terms were significantly negative in each experimental treatment (Table 1), we deemed all FRs to be categorically Type II. Attack rates of *L. raynerae* did not differ significantly between cue-free and *G. affinis* cue treatments (chemical cue: *z* = 0.63, *p* = 0.53; visual cue: *z* = 0.30, *p* = 0.76; both cues: *z* = 0.31, *p* = 0.76), and there was no significant difference within cue treatments (all *p* ≥ 0.44). Handling times of *L. raynerae* also did not vary significantly between cue-free and *G. affinis*-treated waters (chemical cue: *z* = 0.99, *p* = 0.32; visual cue: *z* = 0.32, *p* = 0.75; both cues: *z* = 0.20, *p* = 0.84), and there was, again, no significant difference within cue treatments (all *p* ≥ 0.24). Confidence intervals overlapped amongst all FRs across the entire spectrum of prey supplies, further illustrating similarities in attack rate, handling time and, inversely, maximum feeding rate parameters between different cue treatments (Fig. 1).

**Table 1:** First order terms and significance levels resulting from logistic regression of proportion of prey eaten as a function of prey density, alongside FR parameter estimates across cue treatments with significance levels resulting from the Rogers’ random predator equation in Experiment 1.

Chemical cue	Visual cue	First order term, *p*	*a*, *p*	*h*, *p*
No	No	−0.05, 0.001	0.66, 0.04	0.23, 0.002
Yes	No	−0.06, <0.001	1.24, 0.16	0.35, <0.001
No	Yes	−0.07, <0.001	0.80, 0.02	0.26, <0.001
Yes	Yes	−0.04, 0.005	0.54, 0.03	0.21, 0.005

**Figure 2:**
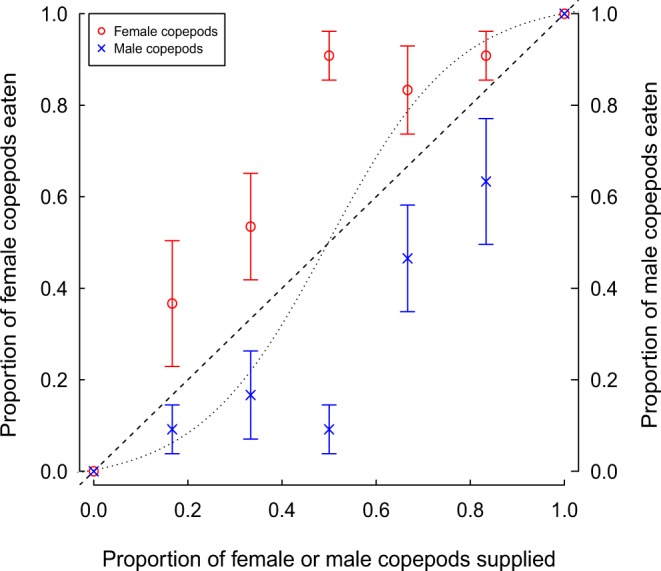
Proportion of female and male *L. raynerae* in diet of *G. affinis* as a function of the proportion supplied. The dashed line indicates the expected value if there was no preferential selection between the two prey types. The dotted sigmoid line represents a hypothetical switching pattern and means are ± standard error (*n* = 4 per group).

In Experiment 2, mosquitofish displayed strong preference for female over male copepods at all prey proportions with the exception of extreme ratios (30:0, 0:30), wherein prey choice was necessarily restricted to one copepod sex (Table 2; Fig. 2). Thus, prey switching did not occur between male and female copepod prey, with preference for female copepods exhibited even when presented at relatively low proportions relative to males. Overall consumption was significantly greater for females than males (*F*_1,54_ = 20.22, *p* < 0.001), and was significantly affected by the proportion of prey available (*F*_6,48_ = 10.89, *p* < 0.001), with greater consumption for a specific prey type exhibited when it was available in higher proportions. There was no significant ‘sex × proportion’ interaction (*F*_6,42_ = 1.01, *p* = 0.44), and thus this interaction was removed from the model. Manly’s *α* preference indices were significantly greater for females, suggesting an overall preference for this prey type (*χ*^2^ = 31.17, *df* = 1, *p* < 0.001; Table 2). Manly’s *α* values were additionally significantly affected by the proportions of prey available (*χ*^2^ = 58.82, *df* = 6, *p* < 0.001), and there was a significant ‘sex × proportion’ interaction (*χ*^2^ = 15.08, *df* = 6, *p* = 0.02), with greater preference for females shown at intermediate prey ratios (Fig. 2).

**Table 2:** Mean untransformed Manly’s *α* preference index values for female or male *L. raynerae* displayed by *G. affinis* across varying proportions (*n* = 4 per treatment).

Proportion supplied	Sex	Manly’s *α* (±SE)
1.00	Female	1.00 (±0.00)
0.83	Female	0.73 (±0.16)
0.67	Female	0.75 (±0.14)
0.50	Female	0.92 (±0.05)
0.33	Female	0.68 (±0.12)
0.17	Female	0.63 (±0.21)
0.00	Female	0.00 (±0.00)
1.00	Male	1.00 (±0.00)
0.83	Male	0.37 (±0.21)
0.67	Male	0.32 (±0.12)
0.50	Male	0.08 (±0.05)
0.33	Male	0.25 (±0.14)
0.17	Male	0.27 (±0.16)
0.00	Male	0.00 (±0.00)

Index values range from 0–1, with 0.5 indicating no preference and values closer to 1 indicating increasing preference.

## Discussion

The identification of measures to understand and forecast invasive species impacts on recipient ecosystems is critical for biodiversity protection and developing proactive management approaches for invasions^34,55^. In our study system, we forecast trait- and density-mediated impacts of a widespread, invasive fish, the mosquitofish *G. affinis*, on an endemic intermediate predator, the calanoid copepod *L. raynerae*. We apply FR^32,33^ and prey switching^42^ approaches experimentally, showing firstly that the feeding magnitude of *L. raynerae* is not significantly affected by either chemical or visual cues of *G. affinis*. Secondly, our study highlights the much higher susceptibility of female over male *L. raynerae* copepods to *G. affinis* predation. Therefore, we show that the potential for invader impact is high, given that the invasive mosquitofish readily consumes and impacts populations of naïve intermediate predators of mosquito larvae, which may affect overall biotic resistance towards mosquito prey. In addition, invader impact may have implications for *L. raynerae* demographics as the copepod exhibits sex-skewed vulnerabilities to the invasive fish. These results are pertinent given that Wasserman *et al*.^41^ showed, conversely, that natural predation on *L. raynerae* by common aquatic insects resulted in lower risk levels for females. Thus, augmented vertebrate predation through *G. affinis* introductions would likely have implications for *L. raynerae* population sex demographics in natural systems, having a further destabilising effect which may reduce population persistence of threatened endemic populations.

Predatory copepods, such as *L. raynerae*, often dominate small aquatic ecosystems which are of high importance for biodiversity in arid environments^56,57^. The small ecosystems which *L. raynerae* dominate function entirely differently to other aquatic systems, and are characterised by restricted higher trophic structuring^52^. Thus, populations within these habitats are especially vulnerable to augmented higher order predation through species introductions^19^. Given the orientation of this copepod to surface waters, vulnerabilities of the species to fish predation may be bolstered by indifferent foraging intensities in the presence of predator cues shown here, coupled with a pronounced association with the upper water column where mosquitofish forage^50^. Biotic contexts such as higher predator risk can have a substantial impact on predator-prey interaction strengths^21,47,58^, but can often be dependent on coevolutionary context^10,27,59,60^. Indeed, invertebrates have been found to be generally responsive to higher predator cues arising from different diets^26^. Such responses frequently reduce predatory impacts exerted upon basal prey by intermediate predators^28^. Here, in contrast, we demonstrate naïveté of *L. raynerae* to unfamiliar predators, as indicated by the recurrence of Type II FRs and similarities in FR parameters (attack rates, handling times) between cue treatments. The exhibited Type II form here corroborates with results of Wasserman *et al*.^61^ and Cuthbert *et al*.^62,63^, where destabilising FRs of *L. raynerae* were also constrained with daphniids and culicids as a basal prey.

In addition to indirect interactions, selectivity by higher-order predators can have direct implications for the demographics of recipient ecosystems^41^. Higher male copepod vulnerability to predation has been recurrently hypothesised due to risks associated with mate-searching and copulation^64,65^. Indeed, Wasserman *et al*.^41^ illustrated that predation of *L. raynerae* by native hexapods is selective towards males due to the processes of copulation. Here, however, we find the opposite in the presence of an invasive higher predator, with high, frequency-independent selectivity demonstrated towards females, which are larger and less motile than males (Cuthbert pers. obs.). The lack of prey switching exhibited here is indicative of an absence of prey refuge for female *L. raynerae* when available in lower proportions, which may have stark implications for demographics and the reproductive success in mature zooplankton populations following invasive fish introductions. The mechanisms of higher-order predatory pressure from fish operate entirely differently from invertebrates; where partial prey consumption is often exhibited by invertebrates, fish consume prey whole^41^. Therefore, the selective tendencies of higher-order fish predation towards females exhibited here may be compounded by the nullification of risk-evasion responses of females when copulating, with copulating pairs perceived, rather, as a single prey unit by fish. This is particularly relevant in light of the extended copulation period of *L. raynerae* and associated reduced instantaneous escape speed^41^. Thus, the introduction of invasive fish may fundamentally alter the demographics of prey populations in aquatic systems ecosystems previously dominated by invertebrates, potentially increasing extinction risk.

## Conclusion

The spread of invasive species continues to circumvent biogeographical barriers and reduce biodiversity, and impacts on recipient communities can be intensified due to naïveté in recipient ecosystems^13,14^. Here, we illustrate, through the coupled use of experimental FR and prey switching approaches, that endemic intermediate predators in insular aquatic ecosystems are naïve to cues from the invasive mosquitofish *G. affinis*, and that selective predation by mosquitofish may affect the population structuring and persistence of native species. Furthermore, *G. affinis* will consume endemic intermediate predators of mosquito larvae that have themselves been suggested for use in mosquito biocontrol^62,63^. The frequency-independent preferences for female copepods demonstrated here by mosquitofish defies the selective preference for male copepods which has been typically posited^64,65^. Thus, the introduction of invasive mosquitofish for vector control could fundamentally shift the dynamics in recipient ecosystems, with effects on intermediate predators that potentially nullify or reverse attempts to control important vector mosquitoes through interguild predation^48^. We advocate that the use of FRs and prey switching offer robust and quantitative insights into the coupled direct and indirect impacts of invasive species on native populations. Prior examinations of such impacts could help to curtail damaging introductions, for instance through ‘classical’ biological control approaches which seek to release non-native agents into novel environments. Further research which incorporates multiple co-existing and interacting invaders alongside native biota would be of additional value in deciphering additive or non-additive trophic interactions within our framework.

## Materials and Methods

### Animal collection and maintenance

Ethical approval for experiments was granted by the animal ethics committee (AEC) within SAIAB (REF# 25/4/1/7/5_2017-14), in accordance with The South African National Standard for the Care and Use of Animals for Scientific Purpose (SANS 10386:2008). *Gambusia affinis* (34.7 ± 1.0 mm) were sourced from irrigation ponds within the Sundays River Valley, Eastern Cape, South Africa (33°26′23.38″S, 25°42′25.67″E) by seine netting in the austral summer 2017. Fish were transported in continuously aerated source water to a controlled environment room at Rhodes University, Grahamstown, maintained at 25.0 ± 1.0 °C and under a 14:10 light:dark regime. Fish were housed in continuously aerated 25 L aquaria containing dechlorinated tapwater and fed on a standard diet of *C. pipiens ad libitum* for at least 12 d prior to experimentation. *Lovenula raynerae* were collected from a pond in Grahamstown (33°16′47.8″S, 26°35′39.8″E), Eastern Cape, South Africa using a 200 μm mesh net and transported in source water to the same laboratory, and kept in 25 L aquaria containing continuously aerated water (matured tapwater and pond water, 50:50 ratio). Mosquito larvae were cultured using egg rafts collected from artificial containers within the Rhodes University campus, identified upon hatching and reared to the desired size class in the same laboratory using a diet of crushed rabbit pellets (Agricol, Port Elizabeth). Both predators were found to feed readily on larval mosquito prey.

### Experimental design

We conducted two experiments to examine the impacts of the invasive fish *G. affinis* on the intermediate predator *L. raynerae*. Both experiments were undertaken in the environment room (25.0 ± 1.0 °C and under a 14:10 light:dark regime) using strained (20 μm), aerated water. In Experiment 1, individual adult male copepods (4.4 ± 0.1 mm) were selected for experimentation following collective starvation for 48 h and provided *C. pipiens* larvae (2.2 ± 0.1 mm) in transparent glass arenas of 5.6 cm diameter containing 80 mL water at five larval densities (2, 4, 8, 16, 32; *n* = 4 per density and treatment). The 80 mL inner experimental arenas were each placed within a larger opaque polypropylene outer arena of 16.5 cm diameter containing 800 mL water. We employed a fully factorial 2 × 2 experimental design with respect to predatory cues of *G. affinis*. Factor 1 comprised chemical cues (present/absent) and Factor 2 visual cues (present/absent). For chemical cues (Factor 1), a 2 L cue accumulation tank was established. In this tank, *G. affinis* were stocked at a density of 0.5 fish L^−1^ and left unfed for 48 h prior following the standard diet. The *G. affinis* treated water (cue water) was then used as the medium within the 80 mL experimental arenas. To implement visual cues (Factor 2), regular water was again used within the experimental arenas, but a single *G. affinis* was placed within the outer 800 mL arena and allowed to move freely, yet unable to consume the *L. raynerae* within the glass inner arena. Mosquito larvae and mosquitofish were added to the inner and outer arenas, respectively, two hours before the addition of the copepod predators and allowed to settle. Following their addition to the inner arena, copepods fed undisturbed for 6 h, after which they were removed and the remaining prey counted to derive those killed. Controls consisted of a replicate of all treatments in the absence of predators in order to constrain background mortality driven by processes outside of predation.

In Experiment 2, adult female and male copepods (female, 4.8 ± 0.1 mm; male, 4.4 ± 0.1 mm) were supplied at seven different ratios (30:0, 25:5, 20:10, 15:15, 10:20, 5:25, 0:30 individuals; *n* = 4 per ratio) to *G. affinis*, which had been starved for 24 h. These ratios reflect the varying proportions of *L. raynerae* in aquatic ecosystems (see Wasserman *et al*.^41^). Experiments were undertaken in arenas of 16.5 cm diameter containing 2 L water from a continuously aerated source. Once introduced, fish fed undisturbed for 3 h, after which they were removed and remaining living copepods counted and sexed. Controls consisted of a replicate at all treatments in the absence of predators.

### Statistical analyses

All statistical analyses were undertaken in R v3.4.2^66^. In Experiment 1, generalised linear models (GLMs) assuming a Poisson error distribution were used to analyse overall prey consumption with respect to ‘chemical cue’, ‘visual cue’ and ‘prey supply’, and their interactions. Non-significant terms and interactions were removed stepwise from the model to facilitate parsimony, with *χ*^2^ used for model simplification *via* analysis of deviance^67^. Functional response (FR) analyses were undertaken using the ‘frair’ package in R^68^. Logistic regression of the proportion of prey consumed as a function of prey density was used to infer FR types. A Type II FR is characterised by a significantly negative first order term, whilst a Type III FR is characterised by a significantly positive first order term followed by a significantly negative second order term^32,33,69,70^. As prey were not replaced as they were consumed, we applied Rogers’ random predator equation for depleting prey densities^69,70^:

1 $${N}_{e}={N}_{0}(1-\exp \,(a\,({N}_{e}h-T)))$$

where *N*_*e*_ is the number of prey eaten, *N*_0_ is the initial density of prey, *a* is the attack constant, *h* is the handling time and *T* is the total experimental period. The Lambert W function was used to enable model fitting^71^. We used the ‘difference method’^70^ to compare attack rate and handling time parameters generated from FRs across treatments. To account for multiplicity, we compared coefficients against Bonferroni-adjusted *p*-values. Functional responses were non-parametrically bootstrapped (*n* = 2000) to generate confidence intervals, allowing the FRs to be considered in population terms^68^.

In Experiment 2, as residuals were overdispersed, GLMs assuming a quasi-Poisson error distribution were used to compare overall prey consumption with respect to ‘sex’ and ‘proportion’, with *F*-tests used for model simplification. Again, non-significant terms and interactions were removed stepwise^67^. Manly’s α^72,73^ assuming no prey replacement was used to determine prey preferences between prey across the varying provision ratios:

2 $${\alpha }_{i}=(\mathrm{ln}(({n}_{i0}-{r}_{i})/{n}_{i0}))/{{\sum }^{}}_{j=1}^{m}(\mathrm{ln}(({n}_{j0}-{r}_{j})/{n}_{j0}))$$

where *a*_*i*_ is Manly’s selectivity index for prey type *i*, *n*_*i*0_ is the number of prey type *i* available at the start of the experiment, *r*_*i*_ is the number of prey type *i* consumed, *m* the number of prey types, *n*_*j0*_ the number of prey type *j* available at the start of the experiment and *r*_*j*_ is the number of prey type *j* consumed. The value of *α*_*i*_ ranges from 0 to 1, with 0 indicating complete avoidance and 1 indicating complete positive selection. In a two-prey system, values of 0.5 are indicative of null preference. Manly’s *α* indices were transformed to reduce extremes^74^ (0 s, 1 s) prior to analysis:

3 $${a}_{t}=({\alpha }_{i}(n-1)+0.5)/n$$

where *α*_*t*_ is the transformed output and *n* is the sample size. Beta regression using the ‘betareg’ package^75^ in R was used to compare Manly’s *α* values between ‘sex’ and ‘proportion’, and their interactions. Akaike’s Information Criterion was used to confirm that models minimised information loss (lower values indicate a better fit).

## Electronic supplementary material

Dataset 1

Dataset 2

## Data Availability

Raw functional response and prey switching data are available in the electronic supplementary material.

## References

[1] Simberloff D (2013). Impacts of biological invasions: what’s what and the way forward. Trends Ecol. Evol..

[2] Seebens H (2017). No saturation in the accumulation of alien species worldwide. Nat. Commun..

[3] Sala OE (2000). Global biodiversity scenarios for the year 2100. Science.

[4] Leprieur F, Beauchard O, Hugueny B, Grenouillet G, Brosse S (2008). Null model of biotic homogenization: a test with the European freshwater fish fauna. Divers. Distrib..

[5] Planty-Tabacchi AM, Tabacchi E, Naiman RJ, Deferrari C, Décamps H (1996). Invasibility of species-rich communities in riparian zones. Conserv. Biol..

[6] Nilsson C, Berggen K (2000). Alterations of Riparian Ecosystems Caused by River Regulation: Dam operations have caused global-scale ecological changes in riparian ecosystems. How to protect river environments and human needs of rivers remains one of the most important questions of our time. BioScience.

[7] Tickner DP, Angold PG, Gurnel LAM, Mountford JO (2001). Riparian plant invasions: hydrogeomorphological control and ecological impacts. Prog. Phys. Geog..

[8] Alexander ME, Kaiser H, Weyl OLF, Dick JTA (2015). Habitat simplification increases the impact of a freshwater invasive fish. Environ. Biol. Fish.

[9] Ricciardi A, Atkinson SK (2004). Distinctiveness magnifies the impact of biological invaders in aquatic ecosystems. Ecol. Lett..

[10] Cox JG, Lima SL (2006). Naïveté and an aquatic–terrestrial dichotomy in the effects of introduced predators. Trends Evol Evol.

[11] Berglund H, Jaremo J, Bengtsson G (2009). Endemism predicts intrinsic vulnerability to nonindigenous species on islands. Am. Nat..

[12] Paolucci EM, MacIsaac HJ, Ricciardi A (2013). Origin matters: alien consumers inflict greater damage on prey populations than do native consumers. Divers. Distrib..

[13] Salo P, Korpimäki E, Banks PB, Nordström M, Dickman CR (2007). Alien predators are more dangerous than native predators to prey populations. Proc. R. Soc. Lond. B. Biol. Sci..

[14] Polo-Cavia N, Gonzalo A, López P, Martín J (2010). Predator recognition of native but not invasive turtle predators by naïve anuran tadpoles. Animal Behav..

[15] Li Y, Ke Z, Wang S, Smith GR, Liu X (2011). An exotic species is the favorite prey of a native enemy. PLoS ONE.

[16] Alvarez-Blanco P, Caut S, Cerdá X, Angulo E (2017). Native predators living in invaded areas: responses of terrestrial amphibian species to an Argentine ant invasion. Oecologia.

[17] Cuthbert RN, Dickey JWE, McMorrow C, Laverty C, Dick JTA (2018). Resistance is futile: lack of predator switching and a preference for native prey predict the success of an invasive prey species. R. Soc. Open Sci..

[18] Mack RN (2000). Biotic invasions: causes, epidemiology, global consequences, and control. Ecol. Appl..

[19] Dalu T, Wasserman RJ, Dalu MTB (2017). Agricultural intensification and drought frequency increases may have landscape-level consequences for ephemeral ecosystems. Glob. Change Biol..

[20] Haiahem D (2017). Impact of eastern mosquitofish, *Gambusia holbrooki*, on temporary ponds: insights on how predation may structure zooplankton communities. Zool. Ecol..

[21] Barrios-O’Neill D (2014). Fortune favours the bold: a higher predator reduces the impact of a native but not an invasive intermediate higher predator reduces the impact of a native but not an invasive intermediate predator. J. Anim. Ecol..

[22] Abrams PA (1995). Implications of dynamically variable traits for identifying, classifying, and measuring direct and indirect effects in ecological communities. Am. Nat..

[23] Abrams, P. A., Menge, B. A., Mittelbach, G., Spiller, D. & Yodzis, P. The role of indirect effects in food webs in *Food Webs: Integration of Patterns and Dynamics* (eds Polis, A. & Winemiller, K. O.) 371–395 (Chapman & Hall, 1996).

[24] Trussell GC, Ewanchuk PJ, Bertness MD, Silliman BR (2004). Trophic cascades in rocky shore tide pools: distinguishing lethal and nonlethal effects. Oecologia.

[25] Peacor SD, Werner EE (1997). Trait–mediated indirect interactions in a simple food web. Ecology.

[26] Paterson RA (2013). Predator cue studies reveal strong trait-mediated effects in communities despite variation in experimental designs. Anim. Behav..

[27] Carlsson NO, Sarnelle O, Strayer DL (2009). Native predators and exotic prey – an acquired taste?. Front. Ecol. Environ..

[28] Alexander ME, Dick JTA, O’Connor NE (2013). Trait- mediated indirect interactions in a marine intertidal system as quantified by functional responses. Oikos.

[29] Kuebbing SE, Nuñez MA (2015). Negative, neutral, and positive interactions among nonnative plants: patterns, processes, and management implications. Glob. Change Biol..

[30] Wasserman RJ (2016). Using functional responses to quantify interaction effects among predators. Funct. Ecol..

[31] Liu X (2018). More invaders do not result in heavier impacts: The effects of non-native bullfrogs on native anurans are mitigated by high densities of non-native crayfish. J. Anim. Ecol..

[32] Solomon ME (1949). The natural control of animal populations. J. Anim. Ecol..

[33] Holling CS (1959). Some characteristics of simple types of predation and parasitism. Can. Entomol..

[34] Dick JTA (2014). Advancing impact prediction and hypothesis testing in invasion ecology using a comparative functional response approach. Biol. Invasions.

[35] Jeschke JM, Kopp M, Tollrian R (2004). Consumer-food systems: why type I functional responses are exclusive to filter feeders. Biol. Rev. Camb. Philos. Soc..

[36] Barrios-O’Neill D (2016). On the context-dependent scaling of consumer feeding rates. Ecol. Lett..

[37] Wasserman RJ (2016). Emergent effects of structural complexity and temperature on predator-prey interactions. Ecosphere.

[38] Mowles SL, Rundle SD, Cotton PA (2011). Susceptibility to predation affects trait-mediated indirect interactions by reversing interspecific competition. PLoS ONE.

[39] Murdoch WW, Oaten A (1975). Predation and population stability. Adv. Ecol. Res..

[40] Hughes RN, Croy MI (1993). An experimental analysis of frequency-dependent predation (switching) in the 15-spined stickleback. Spinachia spinachia. J. Anim. Ecol..

[41] Wasserman RJ (2018). Sacrificial males: the potential role of copulation and predation in contributing to copepod sex-skewed ratios. Oikos.

[42] Murdoch WW (1969). Switching in general predators: experiments on predator specificity and stability of prey populations. Ecol. Monogr..

[43] Pyke GH (2005). A review of the biology of *Gambusia affinis* and *Gambusia holbrooki*. Rev. Fish Biol. Fish.

[44] Lowe, S., Browne, M, Boudjelas, S. & De Poorter, M. *100 of the world’s worst invasive alien species: a selection from the Global Invasive Species Database*. (Invasive Species Specialist Group, 2000).

[45] Pyke GH, White AW (2000). Factors influencing predation on eggs and tadpoles of the endangered green and golden bell frog *Litoria aurea* by the introduced plague minnow *Gambusia holbrooki*. Aust. Zoo..

[46] Richard J (2002). An observation of predation of a metamorph common eastern froglet (*Crinia signifera*) by the plague minnow (*Gambusia holbrooki*). Herpetofauna.

[47] Pyke GH (2008). Plague minnow or mosquito fish? A review of the biology and impacts of introduced *Gambusia* species. Ann. Rev. Ecol. Evol. Syst..

[48] Hoy JB, Kauffman EE, O’Berg AG (1972). A large-scale test of *Gambusia affinis* and chlorpyrifos for mosquito control. Mosq. News.

[49] Azevedo-Santos VM, Vitule JRS, Pelicice FM, García-Berthou E, Simberloff D (2017). Nonnative Fish to Control *Aedes* Mosquitoes: A Controversial, Harmful Tool. BioScience.

[50] Mansfield S, McArdle BH (1998). Dietary composition of *Gambusia affinis* (Family: Poeciliidae) populations in the northern Waikato region of New Zealand. N. Z. J. Mar. Freshw. Res..

[51] Dussart, B. H. & Defaye, D. *Introduction to the Copepoda. Guides to the Identification of the Microinvertebrates of the Continental Waters of the World* (Backhuys Publishers, 2001).

[52] Dalu T, Wasserman RJ, Froneman PW, Weyl OLF (2017). Trophic isotopic carbon variation increases with pond’s hydroperiod: Evidence from an Austral ephemeral ecosystem. Sci. Rep..

[53] Poizat G, Crivelli AJ (1997). Use of seasonally flooded marshes by fish in a Mediterranean wetland: timing and demographic consequences. J. Fish Biol..

[54] Cucherousset J, Paillisson J-M, Carpentier A, Chapman LJ (2007). Fish emigration from temporary wetlands during drought: the role of physiological tolerance. Fundam. Appl. Limnol..

[55] Dick JTA (2017). Invader Relative Impact Potential: a new metric to understand and predict the ecological impacts of existing, emerging and future invasive alien species. J. Appl. Ecol..

[56] Brendonck L, De Meester L (2003). Egg banks in freshwater zooplankton: evolutionary and ecological archives in the sediment. Hydrobiologia.

[57] O’Neill BJ, Thorp JH (2014). Untangling food-web structure in an ephemeral ecosystem. Freshw. Biol..

[58] Alexander ME, Dick JTA, Weyl OLF, Robinson TB, Richardson DM (2014). Existing and emerging high impact invasive species are characterized by higher functional responses than natives. Biol. Lett..

[59] Wisenden BD, Millard MC (2001). Aquatic flatworms use chemical cues from injured conspecifics to assess predation risk and to associate risk with novel cues. Anim. Behav..

[60] Sih A (2010). Predator-prey naïveté, antipredator behaviour, and the ecology of predator invasions. Oikos.

[61] Wasserman RJ, Alexander ME, Barrios-O’Neill D, Weyl OLF, Dalu T (2016). Using functional responses to assess predator hatching phenology implications for pioneering prey in arid temporary pools. J. Plank. Res..

[62] Cuthbert, R. N. *et al*. Calanoid copepods: an overlooked tool in the control of disease vector mosquitoes. *J. Med. Entomol*. Online (2018). 10.1093/jme/tjy132 30085266

[63] Cuthbert Ross N., Dalu Tatenda, Wasserman Ryan J., Coughlan Neil. E., Callaghan Amanda, Weyl Olaf L.F., Dick Jaimie T.A. (2018). Muddy waters: Efficacious predation of container-breeding mosquitoes by a newly-described calanoid copepod across differential water clarities. Biological Control.

[64] Kiørboe T (2006). Sex, sex-ratios, and the dynamics of pelagic copepod populations. Oecologia.

[65] Gusmão LFM, McKinnon AD (2009). Sex ratios, intersexuality and sex change in copepods. J. Plankt. Res..

[66] R Core Team *R: A language and environment for statistical computing*. (R Foundation for Statistical Computing, 2017).

[67] Crawley, M. J. *The R Book* (John Wiley & Sons, 2007).

[68] Pritchard DW, Paterson RA, Bovy HC, Barrios-O’Neill D (2017). Frair: an R package for fitting and comparing consumer functional responses. Methods Ecol. Evol..

[69] Trexler JC, McCulloch CE, Travis J (1988). How can the functional response best be determined?. Oecologia.

[70] Juliano, S. A. Nonlinear curve fitting: predation and functional response curves in *Design and Analysis of Ecological Experiments* (eds Scheiner, S. M. & Gurevitch, J.) 159–182 (Oxford University Press, 2001).

[71] Bolker, B. M. *emdbook: Ecological Models and Data in R*. (Princeton University Press, 2008).

[72] Manly BFJ (1974). A model for certain types of selection experiments. Biomet..

[73] Chesson J (1983). The estimation and analysis of preference and its relationship to foraging models. Ecology.

[74] Smithson M, Verkuilen J (2006). A Better Lemon Squeezer? Maximum-Likelihood Regression with Beta-Distributed Dependent Variables. Psych. Methods.

[75] Cribari-Neto F, Zeileis A (2010). Beta regression in R. J. Stat. Softw..

